# Supplementary material for: Expression of Oncogenic Drivers in 3D Cell Culture Depends on Nuclear ATP Synthesis by NUDT5
Source: Cancers (Basel). 2019 Sep 10;11(9):1337. doi: 10.3390/cancers11091337 (PMC6770457; doi:10.3390/cancers11091337)
Supplement: Supplementary file 1 [file cancers-11-01337-s001.pdf]

Supplementary Figures

Figure S1

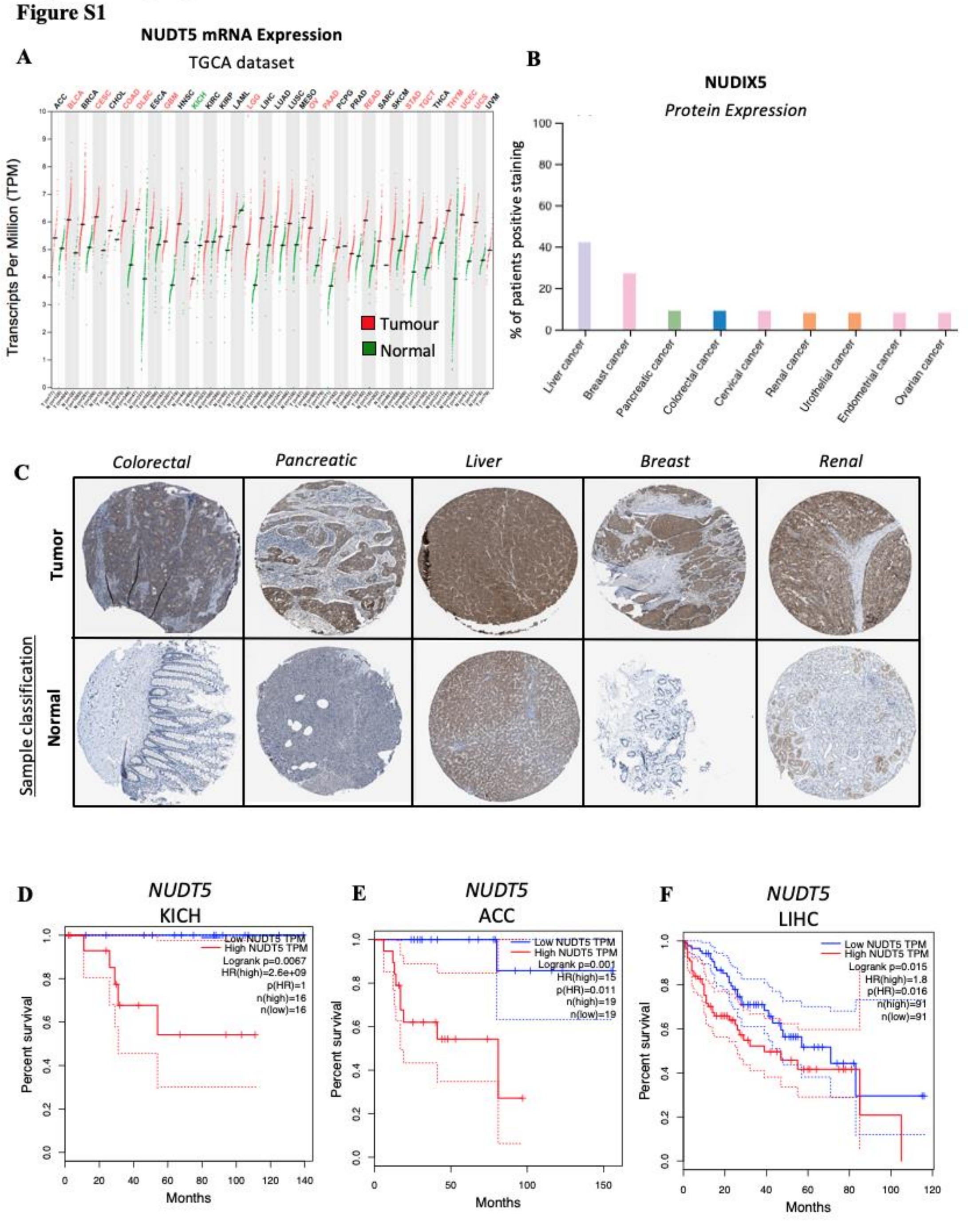

Figure S1 continued

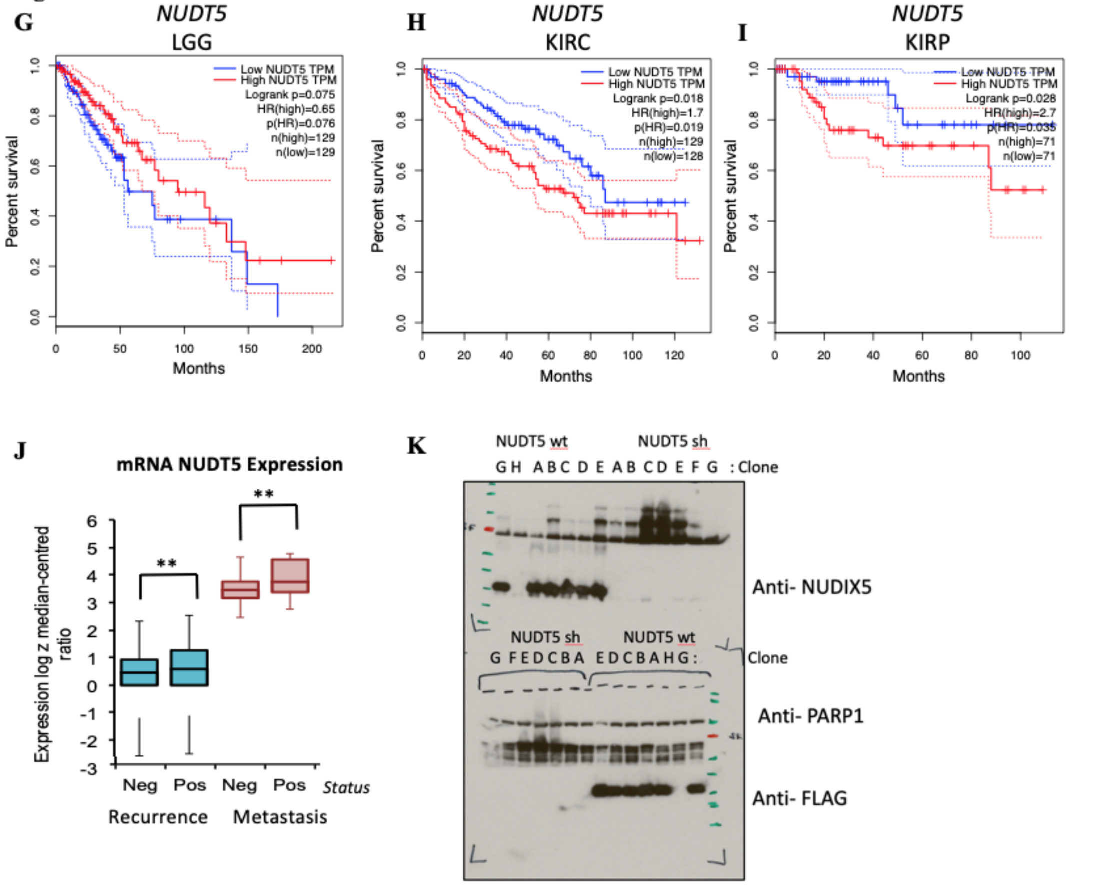

Figure S1: *NUDT5* is indicative of poor prognosis and aggressive cancer growth. (**A**) *NUDT5* mRNA expression across full TGCA database, data for tumour (red) and normal (green) tissue from the same tissue origin are shown using TGCA patient datasets with GEPIA interactive webserver [61]. Datasets where *NUDT5* mRNA levels are elevated in tumour versus normal as highlighted in red, significantly higher in normal versus tumour are marked in green. Abbreviations of the tumour datasets are given in Table S1. (**B**) The percentage of positive staining for NUDT5 protein expression in cancer datasets (Human Protein Atlas database [62]). (**C**) Representative histological staining of NUDT5 in tumour versus normal patient samples from colorectal, pancreatic, liver, breast and renal samples (Human Protein Atlas database [62]). Metadata regarding each of the images are given in Table S2. Kaplan Meyer survival curves stratifying patients based on *NUDT5* mRNA expression in (**D**) Kidney chromophobe (KICH). (**E**) Adrenalcorticocal carcinoma (ACC). **(F)** Liver hepatocellular carcinoma (LIHG) (**G**) Brain lower grade carcinoma (LGG) **(H)** Kidney renal clear cell carcinoma (KIRC) (**I**) Kidney renal papillary cell carcinoma (KIRP) were carried out using TGCA database with GEPIA interactive webserver [61]. (**J**) Recurrence and metastasis analysis in *NUDT5* mRNA high expressing patient cohorts from BRCA dataset shown in Figure S1A. (K) Full western blot of NUDT5, PARP1 and FLAG protein levels in NUDT5^{RES} and NUDT5^{KD} cell clones.

Figure S2

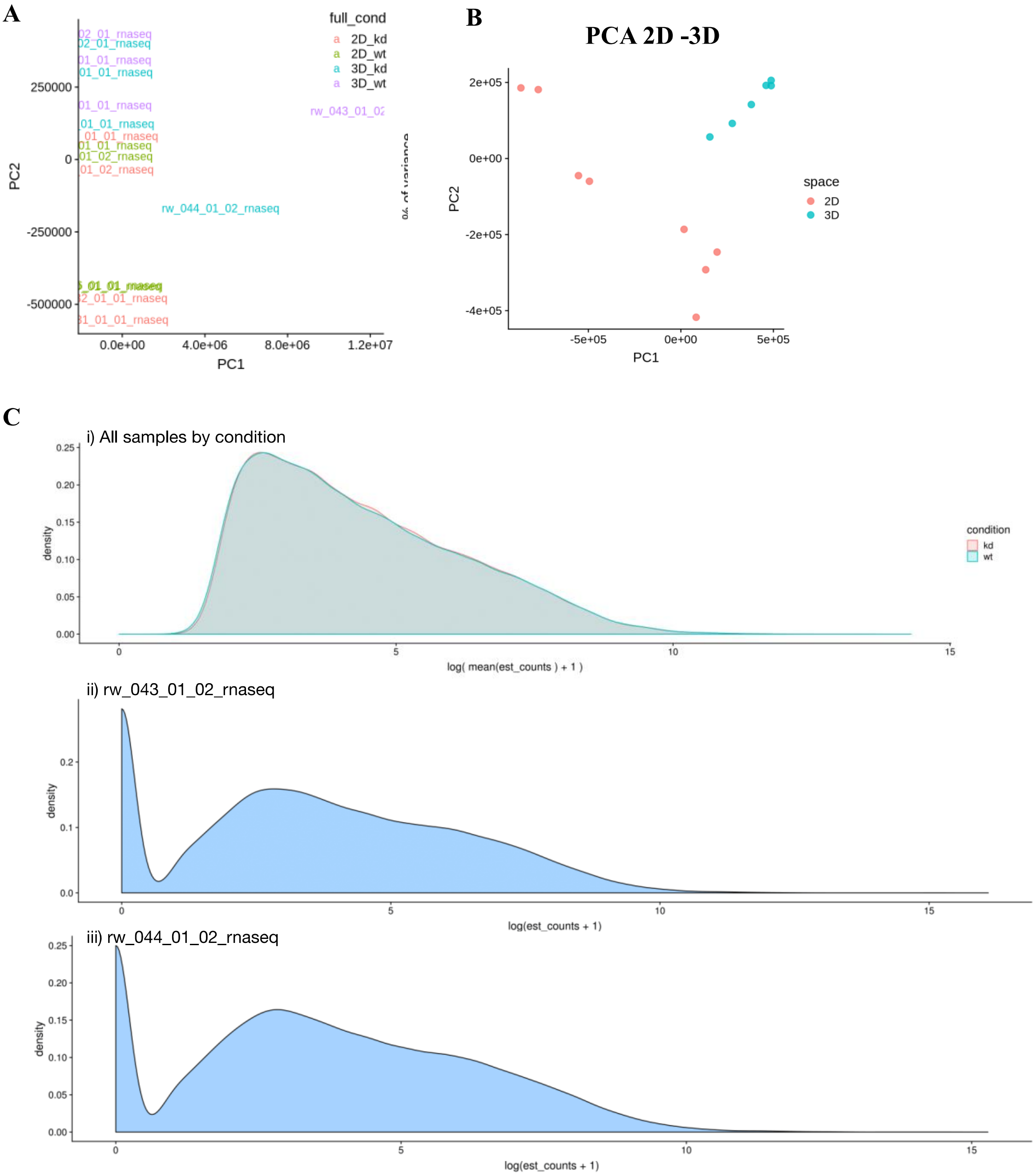
A
full_cond
2D_kd
2D_wt
3D_kd
3D_wt
rw_043_01_02
rw_044_01_02_rnaseq
PC2
PC1
250000
0
-250000
-500000
0.0e+00
4.0e+06
8.0e+06
1.2e+07
B
PCA 2D -3D
space
2D
3D
2e+05
0e+00
-2e+05
-4e+05
-5e+05
0e+00
5e+05
C
i) All samples by condition
condition
kd
wt
density
log(mean(est_counts) + 1)
ii) rw_043_01_02_rnaseq
log(est_counts + 1)
iii) rw_044_01_02_rnaseq
log(est_counts + 1)

Figure S2 continued

D

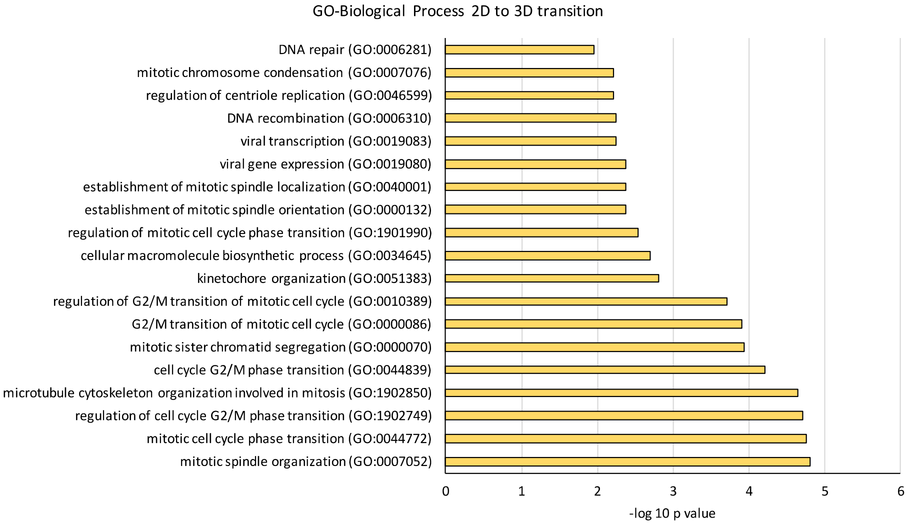

E

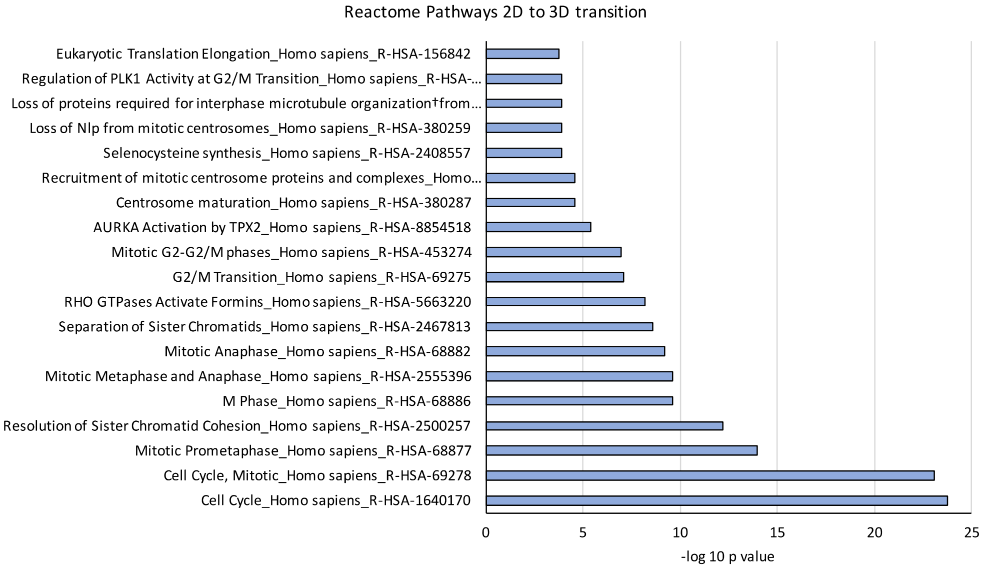

F

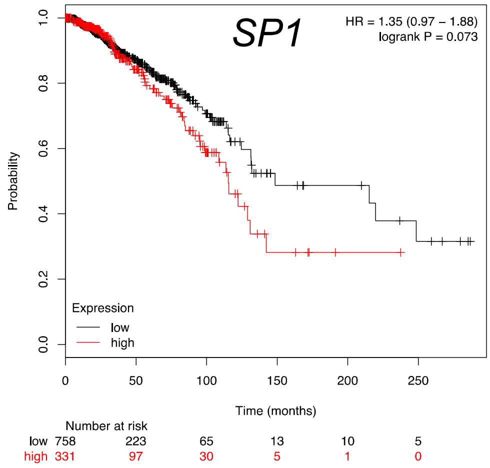

G

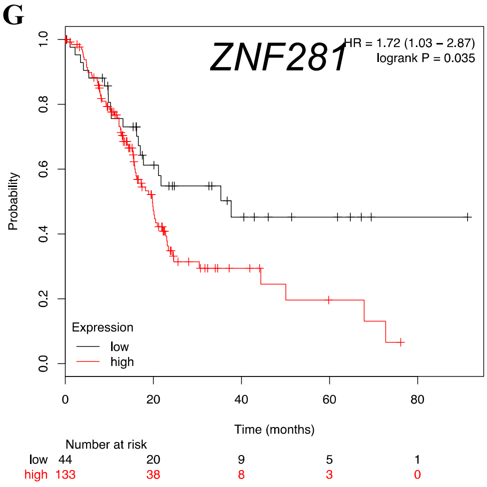

Figure 2. Analysis of the gene expression changes in 3D compared to 2D culture conditions. **(A)** PCA analysis of all RNA samples (Table S3) from T47D cells cultured in 2D and 3D conditions. **(B)** PCA analysis of RNA samples from T47D cells highlighted based on culture conditions. **(C)** Distribution of counts based on all samples (i) or highlighting samples rw_043_01_02_rnaseq (ii) and rw_044_01_02_rnaseq (iii). **(D)** GO-Biological process enrichment analysis showing the top 20

differentially expressed gene classes from 2D and 3D cultured cells. A full list of significant terms is given in Table S4. (**E**) Reactome enrichment analysis of showing top 20 differentially expressed gene classes from 2D and 3D cultured cells. A full list of significant terms is given in Table S5. (**F**) Kaplan Meyer survival curves stratifying patients based on *SP1* expression in breast cancer and on *ZNF281* (**G**) in cervical cancer.

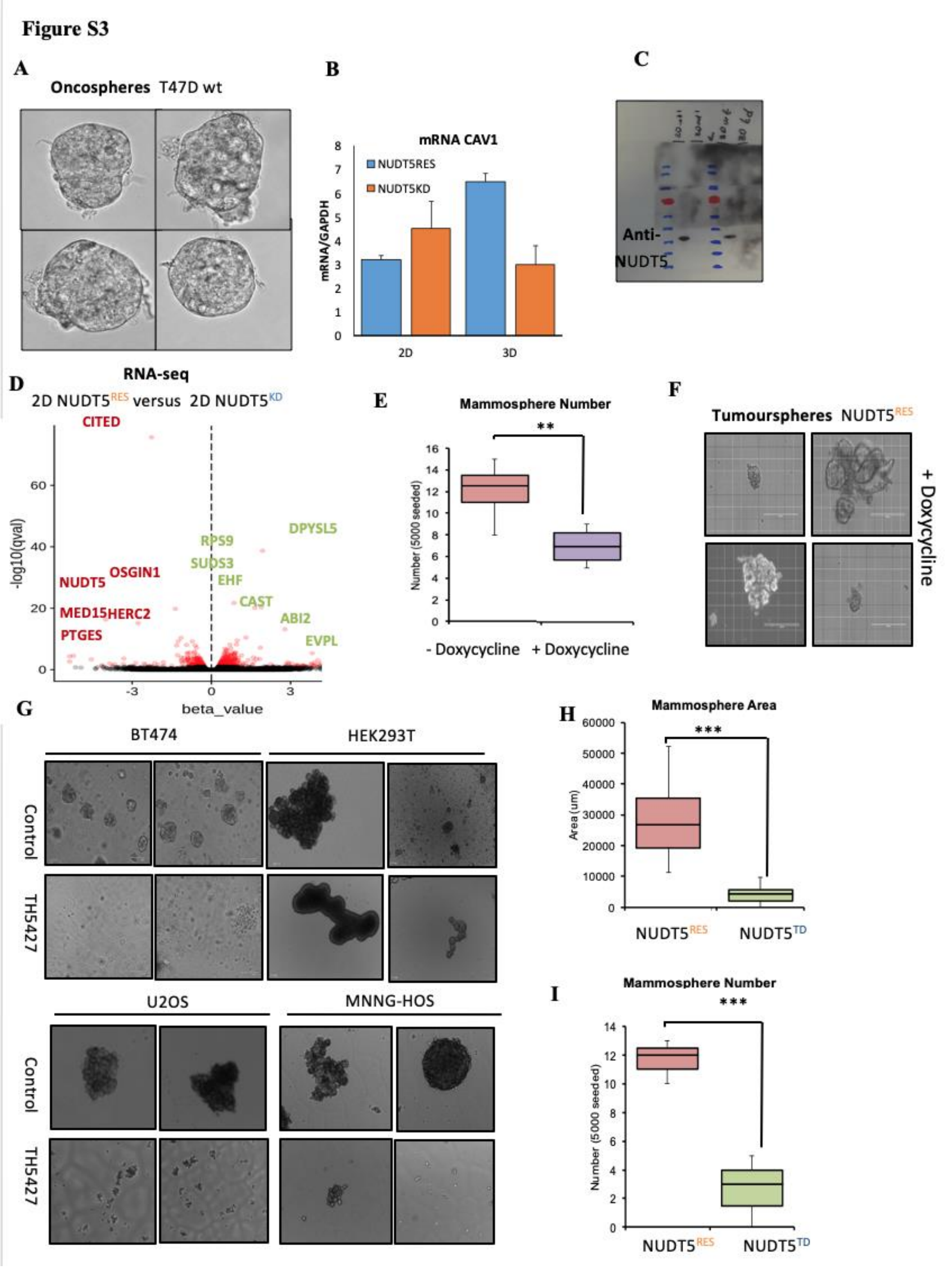

Figure S3: Characterisation of the gene expression changes which dependent on NUDT5 in 3D cell culture.

(**A**) Representative images of oncospheres in T47D^{wt} cells. (**B**) Relative mRNA expression of *CAV1* in NUDT5^{RES} versus NUDT5^{WT} cells grown in 2D and 3D conditions (**C**) Full western blot showing protein expression levels of NUDT5 in NUDT5^{res} (WT) or NUDT5^{KD} (KD) cell lines grown in 2D or 3D (oncosphere) conditions. (**D**) Volcano plot showing the differential gene expression data in NUDT5^{RES} and NUDT5^{KD} cell lines cultured in 2D conditions. The position of *NUDT5, PTGES, CAST, CITED* and other genes named within the main body of text are shown. **(E)** Oncosphere number of NUDT^{RES} cell lines grown in the presence or absence of doxycline; data are mean +/-SEM. (**F**) Representative images of NUDT^{RES} cell lines grown in the presence of doxycline. (**G**) Representative images of oncospheres grown with HEK293T, BT474, U2OS and MNNG-HOS cell lines in the presence or absence of TH5427. Oncosphere area (**H**) and number (**I**) of NUDT5^{RES} versus NUDT5^{TD} cell lines; data are shown as mean +/-SEM.

Figure S4

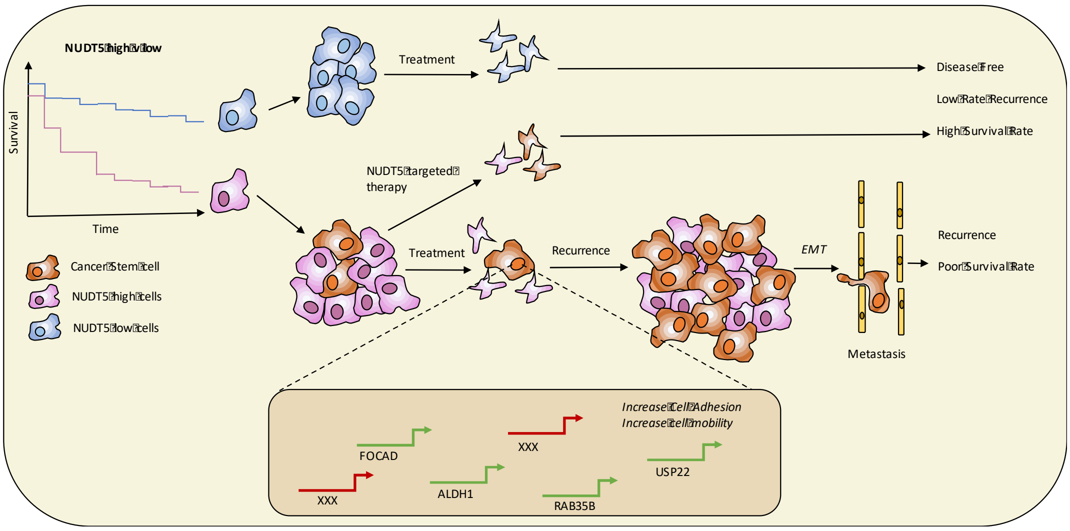

Figure S4: Model for the role of NUDT5 in tumour progression. Stratifying patients based on the expression levels of *NUDT5* predicts a poorer prognosis based on the findings that NUDT5 activity is essential for key tumour drivers, including genes involved in CSC and EMT. This may enable more targeted selection of patients for drastic therapies.

Figure S5

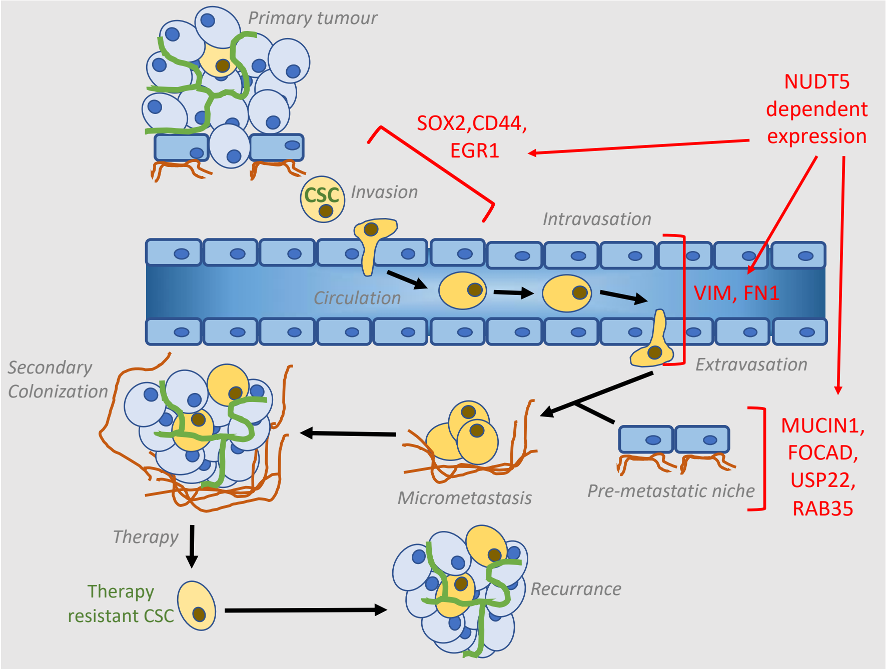

Figure S5: Model for the driving role of NUDT5 expression metastasis.

Diagram depicting the processes during metastasis and the genes expressed during that stage, which are dependent on the ATP generating activities of NUDT5.

Table S1: Abbreviations for TGCA cancer types.

TCGA	Detail
ACC	Adrenocortical carcinoma
BLCA	Bladder Urothelial Carcinoma
BRCA	Breast invasive carcinoma
CESC	Cervical squamous cell carcinoma and endocervical adenocarcinoma
CHOL	Cholangio carcinoma
COAD	Colon adenocarcinoma
DLBC	Lymphoid Neoplasm Diffuse Large B-cell Lymphoma
ESCA	Esophageal carcinoma
GBM	Glioblastoma multiforme
HNSC	Head and Neck squamous cell carcinoma
KICH	Kidney Chromophobe
KIRC	Kidney renal clear cell carcinoma
KIRP	Kidney renal papillary cell carcinoma
LAML	Acute Myeloid Leukemia
LGG	Brain Lower Grade Glioma
LIHC	Liver hepatocellular carcinoma
LUAD	Lung adenocarcinoma
LUSC	Lung squamous cell carcinoma
MESO	Mesothelioma
OV	Ovarian serous cystadenocarcinoma
PAAD	Pancreatic adenocarcinoma
PCPG	Pheochromocytoma and Paraganglioma
PRAD	Prostate adenocarcinoma
READ	Rectum adenocarcinoma
SARC	Sarcoma
SKCM	Skin Cutaneous Melanoma
STAD	Stomach adenocarcinoma
TGCT	Testicular Germ Cell Tumors
THCA	Thyroid carcinoma
THYM	Thymoma
UCEC	Uterine Corpus Endometrial Carcinoma
UCS	Uterine Carcinosarcoma
UVM	Uveal Melanoma

Table S2: IDs from Human Protein Atlas

Tissue Type	Classification	Antibody ID		Patent ID	
Colorectal	Cancer	HPA019827	Colon (T-67000)	2151	https://www.proteinatlas.org/ENSG00000165609-NUDT5/pathology/tissue/colorectal+cancer#img
Pancreatic	Cancer	HPA019827	Pancreas (T-59000)	3614	https://www.proteinatlas.org/ENSG00000165609-NUDT5/pathology/tissue/pancreatic+cancer#img
Liver Cancer	Cancer	HPA019827	Liver (T-56000)	2766	https://www.proteinatlas.org/ENSG00000165609-NUDT5/pathology/tissue/liver+cancer#img
Breast	Cancer	HPA019827	Breast (T-04000)	2428	https://www.proteinatlas.org/ENSG00000165609-NUDT5/pathology/tissue/breast+cancer#img
Kidney	Cancer	HPA019827	Kidney (T-71000)	3533	https://www.proteinatlas.org/ENSG00000165609-NUDT5/pathology/tissue/renal+cancer#img
Colorectal	Normal	HPA019827	Colon (T-67000)	1857	https://www.proteinatlas.org/ENSG00000165609-NUDT5/tissue/colon#img
Pancreatic	Normal	HPA019827	Pancreas (T-59000)	2032	https://www.proteinatlas.org/ENSG00000165609-NUDT5/tissue/pancreas#img
Liver Cancer	Normal	HPA019827	Liver (T-56000)	3222	https://www.proteinatlas.org/ENSG00000165609-NUDT5/tissue/liver#img
Breast	Normal	HPA019827	Breast (T-04000)	3544	https://www.proteinatlas.org/ENSG00000165609-NUDT5/tissue/breast#img
Kidney	Normal	HPA019827	Kidney (T-71000)	3229	https://www.proteinatlas.org/ENSG00000165609-NUDT5/tissue/kidney#img

Table S3: RNA-seq Sample Information

Number	Sample	Space	Condition	full_cond	sequencing
1	rw_026_01_01_rnaseq	2D	wt	2D_wt	06/27/18
2	rw_030_01_01_rnaseq	2D	wt	2D_wt	06/27/18
3	rw_031_01_01_rnaseq	2D	kd	2D_kd	06/27/18
4	rw_032_01_01_rnaseq	2D	kd	2D_kd	06/27/18
5	rw_039_01_01_rnaseq	3D	wt	3D_wt	07/24/18
6	rw_039_02_01_rnaseq	3D	wt	3D_wt	07/24/18
7	rw_040_01_01_rnaseq	3D	kd	3D_kd	07/24/18
8	rw_040_02_01_rnaseq	3D	kd	3D_kd	07/24/18
9	rw_041_01_01_rnaseq	2D	wt	2D_wt	02/06/19
10	rw_042_01_01_rnaseq	2D	kd	2D_kd	02/06/19
11	rw_041_01_02_rnaseq	2D	wt	2D_wt	02/06/19
12	rw_042_01_02_rnaseq	2D	kd	2D_kd	02/06/19
13	rw_043_01_01_rnaseq	3D	wt	3D_wt	02/06/19
14	rw_044_01_01_rnaseq	3D	kd	3D_kd	02/06/19
15	rw_043_01_02_rnaseq	3D	wt	3D_wt	02/06/19
16	rw_044_01_02_rnaseq	3D	kd	3D_kd	02/06/19

Table S4: Curated gene list based on published gene expression signatures linked to 3D culture growth

Reference	Role	Genes
Dontu et al 2017	Adhesion	*HXB,NID,NID2,OSF2*
Ginestier et al 2007, Ben-Porath et al 2010	Pluripotency and stem markers	*ALDH1, CD24,CD44, SOX2, NANOG,POU5F1, EPCAM*
Doyle et al 1998,Pece et al 2010,Callari et al 2016,	Breast Cancer Stem cells	*ABCG2,DNER,ITGA6,ITGA6,*
Dontu et al 2017	Cell cycle	*CCND2*
Dontu et al 2017	Cytoskeleton	*PKD2,WASPIP*
Callari et al 2016, EMT database	EMT	*ACVR1,ADAM17,AGER,AKT1,AMHR2,ANPEP, ANXA1,AR,ATM,AURKA,AXIN1,AXIN2,AXL,BCL2,BCL2L1,BIRC2,BMI1,BMP2,BMP4,BMP7,BOP1,BRAF,C13orf15,CAMK1D,CAV1,CBR1,CD24|CD24L4,CD274,CDH1,CDH13,CDH2,CDH2,CDKN1B,CDKN2A,CDX2,CLDN1,CLDN4,CLU,CMTM8,COL8A1,COL8A2,CSK,CSNK2B,CTBP1, CTGF,CTNNB1,CTNNBIP1,CTNND1,CTSZ,CXCL12,CXCL16,CXCL5,CXCR4,CXCR4,CYR61,DAB2,DAPK1,DDR2,DDX5,DLX4,DNAJB6,ECT2, EDN1,EDN1,EDNRA,EGF,EGFR,EGR1,EIF5A2, ELF5,ELL3,ENG,EPAS1,EPB41L5,EPO,ERBB2IP, ERF,ESR1,ETV4,EZH2,FBLN5,FGF1,FGF2,FGFR2,FHL2,Fibronectin,FLT1,FN1,FOSL1,FOSL1,FOXA1,FOXC1,FOXC2,FOXM1,FOXQ1,FSCN1,FSCN2,GAB2,GATA3,GIPC2,GLRX,GMNN,GREM1,GRIN1,GSK3A,GSK3B,GSN,HAS2,HBEGF,HDAC6,HDGF,HGF,HIF1A,HMGA2,HMGB1,HMGB3,HMOX1,HNF4A,HOXA10,HOXB7,HOXB9, HPGD,HPSE,HRAS,HS3ST3B1,HS6ST2,HSP90*

Reference	Role	Genes
		AA1,HSPA4,HSPB1,HSPB2,ID1,ID1,ID2,IDH1,IDH2,IGF1R,IGFBP3,IL18,IL1B,IL6R,ILK,ITGA5,ITGB1,ITGB3,ITGB4,JAG1,JAG2,JAK2,KCNH1,KHDRBS1,KIT,KL,KLF4,KLF6,KLF8,KLK6,KRAS,KRT19,L1CAM,LAMA1,LAMA5,LATS1,LCN2,LCN2,LEF1,LEFTY1,LEP,LETMD1,LIMA1,LIMS1,LOX,LOXL2,LOXL3,LRP6,LYPD3,MAGED1,MAP2K1,MAP3K3,MAP3K4,MAP3K7,MAPK1,MAPK14,MAPK3,MAPK7,MAPK8,MARVELD3,MCAM,MDK,MET,MGAT3,MKL1,MKL2,MMP13,MMP14,MMP2,MMP3,MMP7,MMP9,MSN,MST1R,MSX2,MTA1,MTA3,MTDH,MUC1,MUC1,MUC16,MUC2,MUC4,MYC,MYCN,N-Cadherin,NCSTN,NDRG1,NF1,NFIC,NFKB1,NOG,NOTCH1,NOTCH2,NRP2,NUMB,OCLN,PAG1,PAK1,PARD3,PARP1,PAX2,PCMT1,PDE4A,PDGFB,PDPN,PHLDA1,PIK3CA,PIN1,PKP3,PLAUR,PLXND1,POSTN,PPARG,PRDX1,PRKCA,PRKCE,PROM1,PRSS8,PTEN,PTGS2,PTHLH,PTK2,PTN,PTPN14,PTPRZ1,RAC1,RAF1,RDX,RGS3,RHOA,RNF111,ROCK1,ROCK2,ROR2,RUNX3,S100A4,SCRIB,SDC1,SDC2,SEMA3E,SEMA4C,SEMA7A,SFTPC,SHH,SIM2,SIX1,SLC39A6,SMAD2,SMAD3,SMAD4,SMAD7,SMAD9,SNAI1,SNAI1,SNAI2,SNAI2,Snail,SNW1,SON,SOX9,SP1,SPP1,SPRR2A,SRC,SRF,ST14,STAT3,STAT5A,STAT5B,STK11,TBX2,TBX20,TBX3,TCF21,TCF3,TCF4,TCF7,TDGF1,TEAD1,TFCP2,TGFA,TGFB1,TGFB1I1,TGFB2,TGFB3,TGFB3,TGFBR1,TGFBR3,TGM2,THBD,TIAM1,TIMP1,TIMP1,TM4SF5,TMPRSS4,TNC,TNF,TP53,TP73,TRPS1,Twist,TWIST1,TXNIP,VCAN,VDR,VEGFA,VIM,VIM,VIM,VSNL1,VTN,VWCE,WISP2,WISP3,WNT1,WNT3A,WNT5A,WNT6,WT1,WWTR1,YAP1,YBX1,YWHAZ,YY1,ZBTB33,ZEB1,ZEB2,ZFYVE9,ZNF217,ZYX

**Reference**	**Role**	**Genes**
Callari et al 2016	Mammospheres	*ABCA12,ABCG1,ACSS1,ALDH3B2,ASS1,BCL6, C9orf152,CAB39L,CASK,CEACAM6,CFB,CKMT1A,CNN2,COMT,CPEB2,CRABP1,CTSA,CYP2J2,DCLK1,DUSP6,EFCAB4A,ENC1,ETV5,FAM129A,FAM134B,FAM46C,FAM83A,HMGCS1,KIAA1199,KIAA1324,KRT81,KRT86,KYNU,LRG1,LTB,MAOA,MGAT4A,MGP,MUCL1,NUCB2,NUPR1,OAS2,PHGDH,PLEKHA4,PMP22,PON2,PRLR,PROM2,PSAT1,QPRT,RRAGD,S100A16,S100A6,S100A8,S100A9,S1PR3,SAT1,SCD,SELENBP1, SERPINA3,SERPINA5,SLC25A18,SOWAHC,SPRY4,TFF3,TM4SF1,TMEM45A,TNFRSF11B,UBE2L6,VIPR1,*
Dontu et al 2017	Membrane protein up regulated in mammospheres	*ABC1,EMP3,FGFR1,FGFR1,FZD2,GHR,GHR,GPC4,IGF2R,NOTCH3,PDGF,*
Dontu et al 2017	Metalloproteases	*ADAM12,AloE,DCN,LIM,PTOV1,TIMP3,*
Visvader et al 2009	Proliferation	*CCNA2,ERBB2,ERBB2,MKI67,*
Al Hajj et al 2005	Signalling in oncospheres	*FYN,GAP43,GPNMB,IGFBP4,IGFBP7,RARRES, SCGF,SCYA2,*
Dontu et al 2017	Transciption factors in oncospheres	*AEBP1,GAS6,WIG1*

Table S5: Protein complexes alterations in 3D culture.

Term	P-value
c-Src-Muc1 complex	0.024152978
Cohesin complex,	0.024152978
ING1-p300-PCNA complex	0.036010705
EGR-EP300 complex	0.024152978
CAV1-VDAC1-ESR1 complex	0.036010705
SIN3-ING1b complex I	0.104203379
OCT1-SOX2 DNA-protein complex	0.024152978
Skeletal muscle sarcoglycan complex SGC, alpha-beta-gamma-delta	0.047724932
Skeletal muscle sarcoglycan complex SGC, alpha-beta-epsilon-gamma	0.047724932
SIN3-ING1b complex II	0.177711908
Sarcoglycan-sarcospan-syntrophin-dystrobrevin complex	0.104203379
Dystrophin-sarcoglycan-syntrophin complex, skeletal muscle	0.115092208
Sarcoglycan-sarcospan-dystroglycan complex	0.070729783
Skeletal muscle sarcoglycan complex SGC, beta-gamma-delta-zeta	0.047724932
Sarcoglycan-sarcospan-complex SG-SPN	0.059297388
OCT4-SOX2 DNA-protein complex	0.024152978
nNos-Capon-Dexras1 complex	0.036010705
p33ING1b-HDAC1 complex	0.024152978
P2X7 receptor signalling complex	0.136475993
Skeletal muscle sarcoglycan complex SGC, epsilon-beta-gamma-delta	0.047724932
NOS3-CAV1-NOSTRIN complex	0.036010705
UTM-SGCE-DAG1-CAV1-NOS3 complex	0.059297388
TF-FVIIa-FXa-TFPI complex	0.047724932
TRP1-G alpha-11-IP3R3-CAV1 signaling complex	0.047724932
Ternary complex	0.036010705
PCI-PSA-SCG2 complex	0.036010705
Rb-NeuroD1-Ngfi-B complex	0.036010705
p33ING1b-PCNA complex	0.024152978
PAX6-SOX2 DNA-protein complex	0.024152978

Table S6: Gene Ontology Biological Processes altered in 2D to 3D cell culture.

**GO BP 2D to 3D transition**	P-value	Adjusted P-value
mitotic spindle organization (GO:0007052)	4.2248E-09	1.57205E-05
mitotic cell cycle phase transition (GO:0044772)	9.58861 E-09	1.78396E-05
regulation of cell cycle G2/M phase transition (GO:1902749)	1.60795 E-08	1.9944E-05
microtubule cytoskeleton organization involved in mitosis (GO:1902850)	2.41429 E-08	2.24589E-05
cell cycle G2/M phase transition (GO:0044839)	8.30091 E-08	6.17754E-05
mitotic sister chromatid segregation (GO:0000070)	1.86088 E-07	0.000115406
G2/M transition of mitotic cell cycle (GO:0000086)	2.34656 E-07	0.000124736
regulation of G2/M transition of mitotic cell cycle (GO:0010389)	4.16096 E-07	0.000193537
kinetochore organization (GO:0051383)	3.76445 E-06	0.001556393
cellular macromolecule biosynthetic process (GO:0034645)	5.35283 E-06	0.001991787
regulation of mitotic cell cycle phase transition (GO:1901990)	8.56354 E-06	0.002896813
establishment of mitotic spindle orientation (GO:0000132)	1.59435 E-05	0.004237542
establishment of mitotic spindle localization (GO:0040001)	1.59155 E-05	0.004237542
viral gene expression (GO:0019080)	1.52948 E-05	0.004237542
viral transcription (GO:0019083)	2.48221 E-05	0.00577268
DNA recombination (GO:0006310)	2.37732 E-05	0.00577268

regulation of centriole replication (GO:0046599)	2.93516 E-05	0.00606763
mitotic chromosome condensation (GO:0007076)	2.93516 E-05	0.00606763
DNA repair (GO:0006281)	5.72335 E-05	0.011208729
strand displacement (GO:0000732)	7.18101 E-05	0.012145693
establishment of spindle orientation (GO:0051294)	6.66736 E-05	0.012145693
DNA replication (GO:0006260)	7.07418 E-05	0.012145693
establishment of chromosome localization (GO:0051303)	9.52075 E-05	0.015402915
regulation of steroid biosynthetic process (GO:0050810)	0.000120 367	0.01735449
SRP-dependent cotranslational protein targeting to membrane (GO:0006614)	0.000128 254	0.01735449
metaphase plate congression (GO:0051310)	0.000120 367	0.01735449
DNA metabolic process (GO:0006259)	0.000125 41	0.01735449
ciliary basal body-plasma membrane docking (GO:0097711)	0.000130 59	0.01735449
DNA biosynthetic process (GO:0071897)	0.000146 749	0.01839593
chromosome condensation (GO:0030261)	0.000148 314	0.01839593
regulation of chromosome segregation (GO:0051983)	0.000198 688	0.023826771
DNA synthesis involved in DNA repair (GO:0000731)	0.000204 906	0.023826771
cotranslational protein targeting to membrane (GO:0006613)	0.000239 462	0.027001104
centromere complex assembly (GO:0034508)	0.000301 334	0.032978323
mitotic nuclear division (GO:0140014)	0.000312 486	0.033221761
protein targeting to ER (GO:0045047)	0.000427 431	0.040578315

mitotic metaphase plate congression (GO:0007080)	0.000394141	0.040578315
chromosome organization (GO:0051276)	0.000435311	0.040578315
DNA-dependent DNA replication (GO:0006261)	0.000435311	0.040578315
regulation of cell cycle process (GO:0010564)	0.000436209	0.040578315

Table S7: Reactome Pathways 2D to 3D Transition

Reactome 2D to 3D transition	Ove rlap	P-value
Cell Cycle_Homo sapiens_R-HSA-1640170	137/566	1.4819E-27
Cell Cycle, Mitotic_Homo sapiens_R-HSA-69278	119/462	1.38549E-26
Mitotic Prometaphase_Homo sapiens_R-HSA-68877	42/107	2.88621E-17
Resolution of Sister Chromatid Cohesion_Homo sapiens_R-HSA-2500257	38/99	2.23348E-15
M Phase_Homo sapiens_R-HSA-68886	63/268	1.04624E-12
Mitotic Metaphase and Anaphase_Homo sapiens_R-HSA-2555396	48/174	1.14148E-12
Mitotic Anaphase_Homo sapiens_R-HSA-68882	47/173	3.5661E-12
Separation of Sister Chromatids_Homo sapiens_R-HSA-2467813	44/162	1.749E-11
RHO GTPases Activate Formins_Homo sapiens_R-HSA-5663220	35/114	4.94744E-11
G2/M Transition_Homo sapiens_R-HSA-69275	43/173	6.40911E-10
Mitotic G2-G2/M phases_Homo sapiens_R-HSA-453274	43/175	9.42156E-10
AURKA Activation by TPX2_Homo sapiens_R-HSA-8854518	23/72	4.33607E-08
Centrosome maturation_Homo sapiens_R-HSA-380287	23/79	2.90897E-07
Recruitment of mitotic centrosome proteins and complexes_Homo sapiens_R-HSA-380270	23/79	2.90897E-07
Eukaryotic Translation Elongation_Homo sapiens_R-HSA-156842	23/89	2.88694E-06
Selenocysteine synthesis_Homo sapiens_R-HSA-2408557	23/87	1.88809E-06
Loss of Nlp from mitotic centrosomes_Homo sapiens_R-HSA-380259	20/69	1.84413E-06

Loss of proteins required for interphase microtubule organization†from the centrosome_Homo sapiens_R-HSA-380284	20/69	1.84413E-06
Regulation of PLK1 Activity at G2/M Transition_Homo sapiens_R-HSA-2565942	23/87	1.88809E-06
Peptide chain elongation_Homo sapiens_R-HSA-156902	22/84	3.71451E-06
Signaling by Rho GTPases_Homo sapiens_R-HSA-194315	58/367	1.97546E-05
Homologous DNA Pairing and Strand Exchange_Homo sapiens_R-HSA-5693579	14/42	1.08918E-05
SUMO E3 ligases SUMOylate target proteins_Homo sapiens_R-HSA-3108232	23/96	1.1362E-05
Nonsense Mediated Decay (NMD) independent of the Exon Junction Complex (EJC)_Homo sapiens_R-HSA-975956	22/89	1.02607E-05
S Phase_Homo sapiens_R-HSA-69242	26/124	3.92748E-05
Influenza Viral RNA Transcription and Replication_Homo sapiens_R-HSA-168273	27/128	2.51399E-05
Selenoamino acid metabolism_Homo sapiens_R-HSA-2408522	25/111	1.52948E-05
Influenza Life Cycle_Homo sapiens_R-HSA-168255	28/136	2.86652E-05
Viral mRNA Translation_Homo sapiens_R-HSA-192823	21/84	1.34316E-05
Presynaptic phase of homologous DNA pairing and strand exchange_Homo sapiens_R-HSA-5693616	13/39	2.24196E-05
SUMOylation_Homo sapiens_R-HSA-2990846	23/101	2.73971E-05
Influenza Infection_Homo sapiens_R-HSA-168254	29/147	4.77271E-05
Eukaryotic Translation Termination_Homo sapiens_R-HSA-72764	21/87	2.3884E-05
RHO GTPase Effectors_Homo sapiens_R-HSA-195258	42/255	0.000103611
Formation of a pool of free 40S subunits_Homo sapiens_R-HSA-72689	22/96	3.66362E-05
HDR through Homologous Recombination (HRR)_Homo sapiens_R-HSA-5685942	17/64	3.78269E-05
3' -UTR-mediated translational regulation_Homo sapiens_R-HSA-157279	23/106	6.14922E-05

GTP hydrolysis and joining of the 60S ribosomal subunit_Homo sapiens_R-HSA-72706	23/107	7.17141E-05
L13a-mediated translational silencing of Ceruloplasmin expression_Homo sapiens_R-HSA-156827	23/106	6.14922E-05
Cap-dependent Translation Initiation_Homo sapiens_R-HSA-72737	24/114	7.16439E-05
Eukaryotic Translation Initiation_Homo sapiens_R-HSA-72613	24/114	7.16439E-05
Mitotic G1-G1/S phases_Homo sapiens_R-HSA-453279	26/136	0.00019568
G2/M Checkpoints_Homo sapiens_R-HSA-69481	27/150	0.000411326

Table S8: Kegg Pathways 2D to 3D transition

Kegg 2D to 3D	P-value
Homologous recombination	0.00001
Cell cycle	0.00028
Mismatch repair	0.00063
Steroid biosynthesis	0.00093
Valine, leucine and isoleucine degradation	0.00316
Progesterone-mediated oocyte maturation	0.00317
Insulin resistance	0.00362
DNA replication	0.00395
Synthesis and degradation of ketone bodies	0.00899
Cellular senescence	0.00953
Thyroid hormone synthesis	0.01475
Ribosome	0.01998
Fanconi anemia pathway	0.02161
Propanoate metabolism	0.02204
Nucleotide excision repair	0.02333
Base excision repair	0.02585
FoxO signaling pathway	0.02878
Cortisol synthesis and secretion	0.03054
Central carbon metabolism in cancer	0.03054
Nicotinate and nicotinamide metabolism	0.04903
Mucin type O-glycan biosynthesis	0.05633
Pyruvate metabolism	0.05821
Mitophagy	*0.066224
Estrogen signaling pathway	*0.069164
Ovarian steroidogenesis	*0.071377

Table S9: Full transcription factor binding sites motifs

FBS cluster name	Target gene hits	Target gene non-hits	Z-score ▲	Fisher score
C75 - Zinc-coordinating::BetaBetaAlpha-zinc finger	56	3	14.155	12.039
C30 - Zinc-coordinating::BetaBetaAlpha-zinc finger	22	37	12.802	6.851
C28 - Zinc-coordinating::BetaBetaAlpha-zinc finger	35	24	12.671	11.58
C104 - Zinc-coordinating::BetaBetaAlpha-zinc finger	48	11	12.203	10.734
C65 - Zinc-coordinating::BetaBetaAlpha-zinc finger	22	37	11.522	3.411
C88 - Zinc-coordinating::BetaBetaAlpha-zinc finger	40	19	10.755	11.208
C27 - Zinc-coordinating::BetaBetaAlpha-zinc finger	34	25	9.136	8.208
C34 - Zinc-coordinating::BetaBetaAlpha-zinc finger	27	32	8.843	5.076
C129 - Other Alpha-Helix::MADS	7	52	8.746	5.144
C80 - Zinc-coordinating::BetaBetaAlpha-zinc finger	11	48	8.117	6.586
